# The role of the CD8+ T cell compartment in ageing and neurodegenerative disorders

**DOI:** 10.3389/fimmu.2023.1233870

**Published:** 2023-07-28

**Authors:** Eleonora Terrabuio, Elena Zenaro, Gabriela Constantin

**Affiliations:** Department of Medicine, Section of General Pathology, University of Verona, Verona, Italy

**Keywords:** CD8+ T lymphocytes, tissue resident memory CD8+ T cells, neurotoxicity, neurodegenerative diseases, cytotoxicity

## Abstract

CD8+ lymphocytes are adaptive immunity cells with the particular function to directly kill the target cell following antigen recognition in the context of MHC class I. In addition, CD8+ T cells may release pro-inflammatory cytokines, such as tumor necrosis factor-α (TNF-α) and interferon-γ (IFN-γ), and a plethora of other cytokines and chemoattractants modulating immune and inflammatory responses. A role for CD8+ T cells has been suggested in aging and several diseases of the central nervous system (CNS), including Alzheimer’s disease, Parkinson’s disease, multiple sclerosis, amyotrophic lateral sclerosis, limbic encephalitis-induced temporal lobe epilepsy and Susac syndrome. Here we discuss the phenotypic and functional alterations of CD8+ T cell compartment during these conditions, highlighting similarities and differences between CNS disorders. Particularly, we describe the pathological changes in CD8+ T cell memory phenotypes emphasizing the role of senescence and exhaustion in promoting neuroinflammation and neurodegeneration. We also discuss the relevance of trafficking molecules such as selectins, mucins and integrins controlling the extravasation of CD8+ T cells into the CNS and promoting disease development. Finally, we discuss how CD8+ T cells may induce CNS tissue damage leading to neurodegeneration and suggest that targeting detrimental CD8+ T cells functions may have therapeutic effect in CNS disorders.

## Introduction

The central nervous system (CNS) has been previously viewed as an immune-privileged site inaccessible to peripheral immune cells during normal, steady-state conditions (1). The role of neuroinflammation in neurodegenerative disorders, such as Alzheimer’s disease (AD) and Parkinson’s disease (PD), has been disregarded for a long period of time. However, current research has completely redefined the concept of CNS immunity, shifting it from the belief that the brain is an isolated organ, impervious to peripheral immune cells, to the recognition of the key role for immune mechanisms and neuroimmune interactions during physiological and pathological conditions (2–7). Whereas the role of innate immunity, especially microglia, in neurodegeneration was the focus of numerous studies, the involvement of adaptive immune cells, particularly CD8+ T lymphocytes, in neurological disorders was less explored (2, 5, 6, 8–19). The reason for this discrepancy may be due to a heterogeneous and plastic CD8+ T cell compartment, with T cell subsets that have not been yet well characterized in both normal and diseased conditions (7, 20, 21). Indeed, recent studies started to unveil the phenotypic and functional alterations occurring in the subpopulations of CD8+ T lymphocytes in various CNS diseases, but this research area is still in its infancy and many questions remain unanswered (6, 12, 14, 17–19, 22–25). Although CD8+ T lymphocytes are present in significant numbers in the brains of healthy individuals (22), their activity needs to be properly regulated in order to prevent potential detrimental local effects (15). Aging is widely recognized as a major risk factor for the development of neurodegenerative diseases and the aged CNS is characterized by a gradual loss of naïve and memory CD8+ T cells and an exponential increase in the number of transcriptionally altered exhausted and senescent T lymphocytes (20, 23, 26–28). However, the role of aging-induced CD8+ T cell alterations in brain disorders is poorly understood and a comprehensive view of the molecular mechanisms through which CD8+ T lymphocytes contribute to the development of diseases is lacking.

In this review, we discuss the role of CD8+ T lymphocytes in various neuroinflammatory pathologies, including common disorders such as AD, PD, multiple sclerosis (MS), and amyotrophic lateral sclerosis (ALS), as well as rare brain disorders such as limbic encephalitis-induced temporal lobe epilepsy (LE-induced TLE) and Susac syndrome (Sus). We highlight the heterogeneity of CD8+ T cell populations and their multifaced roles and discuss common disease pathways but also how CD8+ T cells may specifically promote aging and the development of neurodegenerative diseases.

## CD8+ T cells origins and differentiation

CD8+ T lymphocytes are adaptive immune cells that arise from the bone marrow and mature in the thymus (26). After the release in the bloodstream, mature naïve CD8+ T cells search for their cognate antigen presented in the context of major histocompatibility complex-I (MHC-I) molecules expressed on the surface of antigen-presenting cells (APC) (29, 30). Upon antigen encounter, naïve CD8+ T lymphocytes become effector cells (31), whose main role is to mediate the apoptosis of the target cell via direct and indirect immune mechanisms, known as T cell-mediated cytotoxicity (32, 33). During this process, conventional T cells first establish contacts with the target cell via FasL-CD95 (FasR) binding, inducing the activation of the caspase cascade and releasing granzymes and perforins to facilitate apoptosis (33). Secondly, they produce pro-inflammatory cytokines, such as tumor necrosis factor-α (TNF-α) and interferon-γ (IFN-γ), which stimulate the expression of MHC-I and FasR molecules on the surface of the target cell, further promoting its death (32, 34–36). After antigen clearance, most effector CD8+ T lymphocytes undergo a controlled apoptosis during the “contraction phase” of the immune response, with only a small fraction of cells surviving as memory CD8+ T cells, providing immune protection from experienced antigens in the circulation and inside the tissues (37). Importantly, memory CD8+ T lymphocytes are maintained throughout lifetime, but their numbers may vary over time and during certain disease conditions (24, 26, 28, 30).

### Naïve CD8+ T cells

**Figure 1:**
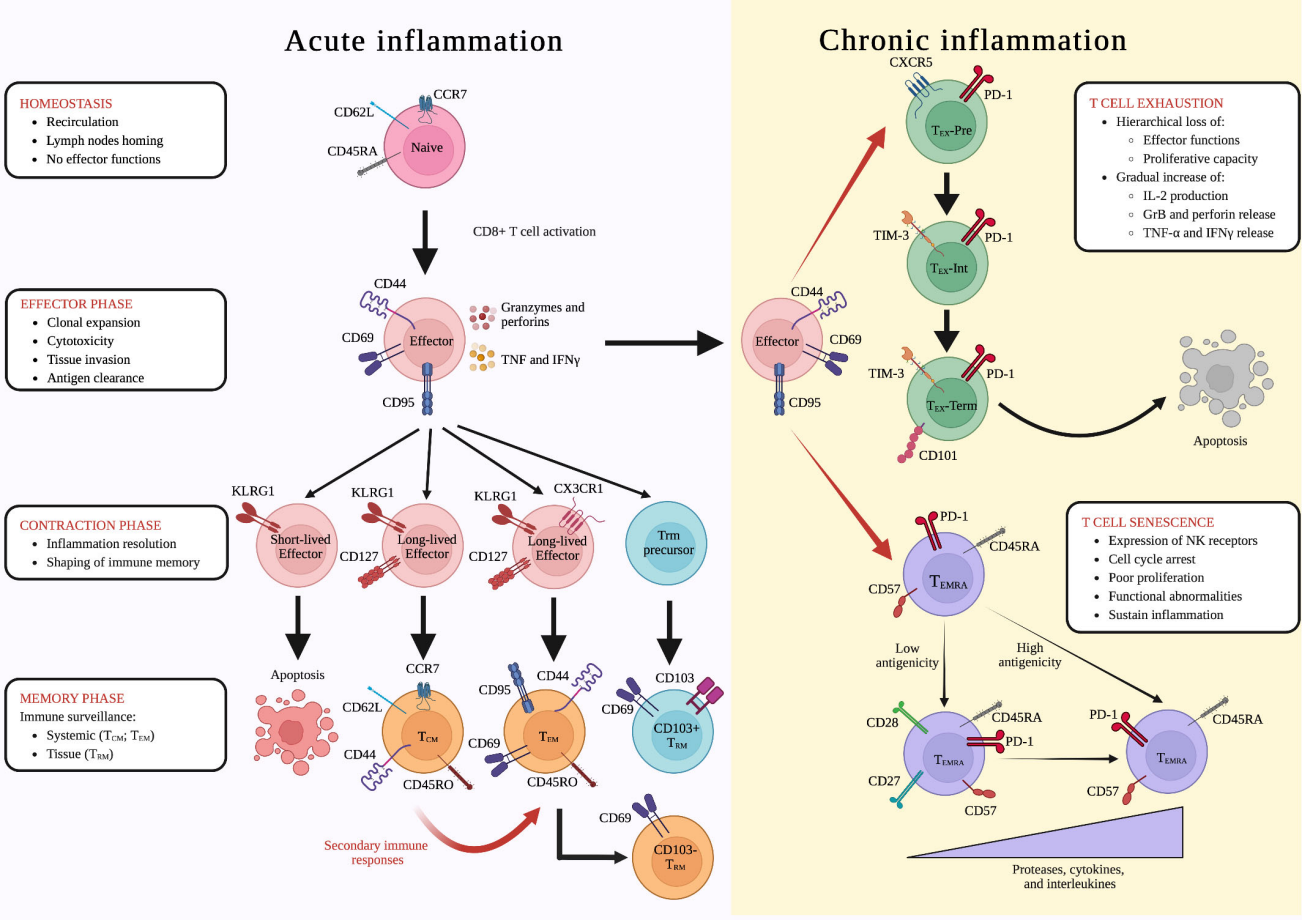
Differentiation of CD8+ T lymphocytes during acute and chronic inflammation. Inflammatory reactions are associated to several conditions including infections, tumors, autoimmune disorders and ageing. During acute inflammatory conditions, such as infections, naïve lymphocytes undergo cell activation becoming effector cells and leading to the “effector phase” of the immune response. Clonally expanded CD8+ T lymphocytes will then invade peripheral tissues, where they can exert cytotoxic functions. After antigen clearance, during the “contraction phase” of the immune response, KLRG1+ CD127- CX3CR1- short-lived effector cells undergo a controlled apoptosis, while long-lived KLRG1+ CD127+ CX3CR1- and KLRG1+ CD127+ CX3CR1+ give origin to central memory (T_CM_) and T effector memory (T_EM_) cells, respectively, shaping immunological memory. Once in the tissues, T_EM_ lymphocytes could also undergo to another differentiation step, leading to the formation of CD69+ CD103- tissue resident memory (T_RM_) cells, whereas the CD69+ CD103+ T_RM_ subset arise from KLRG1- effector T_RM_ precursors. In addition, in case of re-infection with the same antigen, during the so-called “secondary immune response”, T_CM_ lymphocytes are rapidly re-activated, differentiating into T_EM_ cells. This differentiation cascade is altered under chronic immune and inflammatory reactions in which antigen stimulation is prolonged and effector CD8+ T lymphocytes undergo two distinct differentiation steps: **(i)** exhaustion, which is characterized by a hierarchical loss of effector functions and a gradual increase of intrleukin-2 (IL-2) production and granzyme B, perforin, tumor necrosis factor (TNF), and interferon gamma (IFNγ) release (CXCR5+ PD-1+ T_EX_-pre ➔ TIM-3+ PD-1+ T_EX_ -Int ➔ CD101+ TIM-3+ PD-1+ T_EX_-Term); and **(ii)** senescence, during which CD57+ PD-1+ CD45RA+ T_EMRA_ CD8+ T lymphocytes expressing natural killer (NK) receptors undergo cell cycle arrest, poor proliferation, and functional abnormalities, sustaining inflammation. Depending on the strength of antigenic stimulation, T_EMRA_ cells release increased levels of senescent-associated secretory phenotype (SASP)-related proteases, cytokines, and interleukins (CD27+ CD28+ T_EMRA_ ➔ CD27- CD28- T_EMRA_). Created with Biorender.com.

Naïve CD8+ T lymphocytes are mature circulating cells that can acquire various effector functions depending on external clues (38). Their differentiation program is not pre-determined, but is shaped instead by conditions such as inflammatory states and ageing (39). In mice, the naïve phenotype is characterized by the expression of surface markers CD62L (L-selectin) and CD197 (CCR7), while in humans also includes the expression of CD45RA epitope (32, 39, 40) (**Figure 1**). Upon activation, naïve CD8+ T cells lose the expression of homing receptors, initiating a proliferation and differentiation program resulting in an army of effector CD8+ T lymphocytes (31, 40, 41) (**Figure 1**). In addition to the traditional method of identifying cells based on their classical surface markers, a new approach has emerged that focuses on their metabolic traits. Recent research has shown that naïve and memory T cells rely on oxidative phosphorylation and fatty acid oxidation, while effector T cells use aerobic glycolysis and amino acid metabolism to maintain their active state (42, 43).

### Effector CD8+ T cells

In contrast to naïve CD8+ T cells, the fate of effector T lymphocytes is more clearly defined (44, 45). Indeed, these cells are specifically activated and directed toward pathogen-derived or tumor-derived peptides, (29, 46, 47). Effector CD8+ T lymphocytes classically express CD44 and CD69 surface molecules, allowing them to enter peripheral tissues (32, 48, 49) (**Figure 1**). Moreover, they can be identified by the expression of CD95 (FasR) molecule, which contributes to the direct CD8-mediated cytotoxic process, and by the Ki-67 proliferation marker, which is important during clonal expansion (50–52). CD8+ T cells also possess a high cytotoxic potential by secreting various effector and cytotoxic molecules, including granzymes, perforins, IFN-γ, TNF-α and interleukin-2 (IL-2), which enable them to effectively combat infections (32, 33, 53, 54) (**Figure 1**).

Following antigen clearance, a two-tiered contraction occurs in the CD8+ T cell population, defined by the expression of the killer cell lectin-like receptor G member 1 (KLRG1). Short-lived effector KLRG1+ CD127- CD8+ T cells undergo selective apoptosis, whereas effector KLRG1+ CD127+ CD8+ T lymphocytes are preserved and evolve into exKLRG1 long-lived memory T cells, providing immunological memory (37, 41, 44) (**Figure 1**). However, a subset of effector CD8+ T cells, called memory precursors effector (T_MPE_) cells, has been found to lack KLRG1 expression, and this was associated with enhanced survival during the contraction phase and a higher developmental plasticity (37). Accordingly, T_MPE_ cells retain the capability to differentiate in all the subsets of memory cells, playing a critical role in long-term protective immunity (37).

### Memory CD8+ T cells

During the memory phase of the immune response, CD8+ T cells display immunological memory, which enhances their ability to rapidly and effectively respond to previously encountered pathogens, thus safeguarding the body against known threats (55, 56).

The memory compartment of CD8+ T lymphocytes comprises three different cell subsets: (i) T central memory (T_CM_), (ii) T effector memory (T_EM_), and (iii) tissue-resident memory (T_RM_) (55, 57). It is largely known that effector CD8+ T lymphocytes may generate all these memory populations during the “contraction phase” of the immune response and CX3CR1 expression has a key role in this process. Specifically, CX3CR1+ effector T cells appear to preferentially differentiate into T_EM_ cells, while CX3CR1- precursors give rise to T_CM_ cells (37, 58, 59) (**Figure 1**). The different origin of T_CM_ and T_EM_ reflects their phenotypical and functional differences. Similarly to naïve cells, T_CM_ CD8+ T lymphocytes express CD62L and CD197 homing receptors, which are responsible for their recirculating behavior (32, 48, 60). However, T_CM_ cells also express CD44 molecule in mice, and both CD44 and CD45RO epitopes in humans, indicating their memory-like phenotype (39, 61) (**Figure 1**). During the second expansion phase, T_CM_ CD8+ T cells encounter their cognate antigen and differentiate into T_EM_ CD8+ T lymphocytes, losing the expression of homing receptors and migrating into peripheral tissues, where they can release cytotoxic molecules (39, 62) (**Figure 1**). When the antigen is effectively cleared, T_EM_ cells evolve into antigen-specific T_CM_ CD8+ T lymphocytes, and the ultimate goal of these two populations is to provide systemic immunity.

Differently, local protective immune responses are orchestrated by T_RM_ CD8+ T lymphocytes, which have a distinct profile from other CD8+ T cell subsets (59, 63). Notably, T_RM_ CD8+ T cells are not recirculating, but are mainly organized in lymphoid niches close to anatomical and physiological barriers, acting as sentinels and protecting against reinfections (64). While further research is needed to fully understand the microenvironmental signals needed to establish and maintain the population of T_RM_ CD8+ T cells into different peripheral tissues, it is clear that IL-15, IL-7, TGF-β, IL-21, TNF-α, and IL-33 play a crucial role in the formation of this CD8+ T cell subset (21, 65–68). The retention of T_RM_ CD8+ T cells in peripheral tissues is mediated by CD69 and CD103 molecules, which are classically expressed on the surface of both human and murine T_RM_ CD8+ T lymphocytes (22, 37). CD69 inhibits the expression of sphingosine-1-phosphate receptor 1 (S1PR1) molecule, promoting T cell residency, while CD103 integrin, an adhesion receptor for E-cadherin, contributes to CD8+ T cells persistence inside the tissues (69–71). Notably, all T_RM_ CD8+ T lymphocytes express CD103 integrin in lymphoid tissues, but its expression may be lost in non-lymphoid tissues (64, 69). Accordingly, several studies reported the presence of both CD103+ CD69+ and CD103- CD69+ T_RM_ CD8+ T lymphocytes in non-lymphoid tissues, such as liver, brain, gut, skin, and lungs (17, 22, 63, 65, 72–76). Recently, it has been demonstrated that CD103+ and CD103- T_RM_ CD8+ T cells originate from two separate differentiation paths and are characterized by distinct effector functions (22, 37). ExKLRG1 effector CD8+ T cells give rise to CD103- T_RM_ lymphocytes which can be distinguished from CD103+ counterpart due to their cytotoxic potential (37, 65) (**Figure 1**). In contrast, the precursors of CD103+ T_RM_ cells, featuring a lower expression of granzymes and other effector molecules, seem to originate from KLRG1- T_MPE_ CD8+ T lymphocytes (22, 37, 65) (**Figure 1**).

It is now well established that the T_RM_ compartment of CD8+ T lymphocytes upregulates *CXCR6* homing receptor gene, and *ITGA1* gene, encoding CD49a collagen-binding integrin, while downregulating *SELL* and *CX3CR1* genes, encoding for CD62L and CX3CR1 molecules, respectively (17, 57, 77). Moreover, *Runx3*, *Notch*, *Bhlhe40*, *Blimp1* and its homolog *Hobit*, and the AP-1 family members, including *Jun*, *Junb*, *Jund*, *Fos*, *Fosb*, and *Batf* have been identified as crucial transcription factors (TFs) in the regulation of T_RM_ cells formation (59). Additionally, TFs induced by interferon signaling, such as *Stat1*, *Irf1*, *Irf7*, and *Irf9*, or related to the NF-κB signaling pathway, including *Bcl3*, *Rela*, *Relb*, *Rel*, and *Nfkb2* are enriched in T_RM_ T lymphocytes, adding further markers for this CD8+ T cell population (59). Altogether, these data highlight the heterogenicity and complexity within the T_RM_ compartment of the CD8+ T cell population.

### Exhausted CD8+ T cells

All stages of the immune response and their players are perfectly coordinated and functioning under acute inflammatory states, when the immune reaction successfully clears antigens. However, persistent antigen stimulation leads to chronic inflammation, disrupting this harmoniously synchronized mechanism (78, 79). Effector T cells in this condition become dysfunctional, undergoing exhaustion, resulting in poor effector functions and reduced proliferative potential (78, 79) (**Figure 1** ; **Table 1**). Importantly, T cell exhaustion is not just an alteration of cell phenotype and functions, but also represents a distinct differentiation state, with different characteristics compared to the memory features (24, 81). Exhausted (T_EX_) CD8+ T cells maintain the same characteristics under different inflammatory conditions, with a well-defined gene signature, including TCR-signaling related genes such as *Batf*, *Egr2*, *Ezh2*, *Irf4*, *Nfatc1*, *Nfatc2*, *Nr4a1*, *Nr4a2*, and *Nr4a3* (94–96), confirming that continuous exposure to persistent antigens is a key factor in T cell exhaustion, whereas short antigen exposures lead to exhaustion recovery (80) (81) (**Table 1**).

**Table 1:** Main differences between exhausted and senescent CD8+ T lymphocytes.

CD8+ T cell subset	Key features	References
Exhausted	Loss of effector functions and proliferative potential	(78, 79)
	Chronic activation due to prolonged antigen exposure	(80)
	Exhaustion recovery after short antigen exposures	(81)
	Upregulation of inhibitory receptors	(24, 82–85)
	Hypo-functionality	(78, 79)
Senescent	Cell cycle arrest - Replicative senescence	(78, 86–88)
	Increased during chronic inflammation (auto-immune diseases, cancer, ageing)	(88–91)
	Senescence-associated secretory phenotype (SASP)	(87, 92)
	DNA damage response (p16 and p21)	(86, 87)
	Expression of NKR	(93)
	Hyper-functionality	(87, 92)

Hyporesponsive T_EX_ cells are defined by their high surface expression of programmed cell death-1 (PD-1), lymphocyte-activation gene 3 (LAG-3), CD244 (2B4), T-cell immunoglobulin and mucin domain-3 (TIM-3), cytotoxic T-lymphocyte-associated protein-4 (CTLA-4), and CD160 inhibitory receptors (24, 82–85) (**Table 1**). These receptors typically expressed on the surface of T_EX_ CD8+ T bind to a variety of ligands, suggesting that microenvironment clues, such as ligand availability, may regulate the functionality of T_EX_ CD8+ T lymphocytes (78, 79).

T_EX_ lymphocyte compartment is heterogeneous showing three differentiation states: (i) T cell factor 1+ (TCF1+) PD1+ CXCR5+ T_EX_ precursors (T_EX_-Pre), expressing the T-bet TF and showing memory-like features, such as the expression of *Sell*, *Ccr7*, *Id3*, and *Bcl6* genes; (ii) PD1+ TIM-3+ TCF1- Intermediate T_EX_ (T_EX_-Int), not expressing *Zeb1* gene, which encodes for the ZEB-1 TF; and (iii) PD1+ TIM-3+ CD101+ TCF1- terminally differentiated T_EX_ (T_EX_-Term) expressing the TFs ZEB-1, Blimp-1, and Eomesodermin (EOMES) (24, 97, 98) (**Figure 1**). Along the differentiation process, T_EX_-Pre, T_EX_-Int, and T_EX_-Term show a hierarchical decrease of effector activity, marked by mitochondrial dysfunctions, and proliferative capacity, while gradually increasing the expression of inhibitory receptors, ultimately leading to cell death (78, 98, 99) (**Figure 1**). However, T_EX_ lymphocytes do not entirely lack effector functions, exhibiting instead a gradual increase of (i) IL-2 production, (ii) cytotoxicity mediated by granzyme B and perforin, and (iii) release of pro-inflammatory molecules such as IFNγ and TNF-α (78, 79, 98) (**Figure 1** ; **Table 1**).

### Senescent CD8+ T cells

Senescent CD8+ T cells exhibit cell cycle arrest and poor proliferation along with severe functional abnormalities similar to those occurring during T cell exhaustion (78, 87, 88) (**Figure 1** ; **Table 1**). They differentiate from effector CD8+ T cells typically occurring during conditions associated to chronic inflammation, including auto-immune diseases, cancer and ageing (88–91) (**Table 1**). Unlike hypo-functional T_EX_ lymphocytes, senescent CD8+ T lymphocytes continue to secrete a range of factors including proteases such as cathepsins and serine proteases, and cytokines such as CCL5, CCL16, CCL23, TNF-α, IL-29, and IL-18, which in turn may induce IFN-γ production, suggesting a pro-inflammatory senescent-associated secretory phenotype (SASP) (87, 92) (**Table 1**). Moreover, CD45RA molecule is re-express on the surface of these hyper-functional CD8+ T lymphocytes, which are commonly referred as effector memory CD45RA+ (T_EMRA_) cells (91). T_EMRA_ cells are characterized by the simultaneous expression of PD-1, KLRG-1, and CD57 on their surface, which also represent classical phenotypical markers of senescent CD8+ T lymphocytes (82, 91, 100–103) (**Figure 1**). A certain degree of heterogenicity has been also shown among T_EMRA_ lymphocytes. Accordingly, a recent report revealed that effector CD8+ T cells differentiate into CD27- CD28- or CD27+ CD28+ T_EMRA_ cells based on the strength of TCR engagement and the immunogenicity of the tumor antigens (91) (**Figure 1**), while other studies have shown that the lack of CD27 and CD28 surface molecules is associated with the expression of p16 and p21 proteins, causing G1 cell cycle arrest and replicative senescence (86, 87) (**Table 1**).

Senescent CD8+ T cells possess the capability to express natural killer (NK)-associated receptors (NKR), such as NKG2D, NKG2C, NKG2A, and killer immunoglobulin-like receptor (KIR) families, allowing them to be reprogrammed into hyper-functional lymphocytes with the ability to recognize and kill target cells through both TCR and NKR recognition mechanisms (93) (**Figure 1**; **Table 1**). Notably, senescent CD8+ T lymphocytes expressing NKR need to be distinguished from invariant natural killer T (iNKT) cells, which are a subset of natural killer T (NKT) innate immune cells that decrease with age (104, 105). Thus, senescent CD8+ T cells seem to have beneficial as well as detrimental roles during aging, having the potential to retain a broad spectrum of effector functions to kill malignant and infected cells, whilst also having the capacity to induce or sustain autoimmunity and other chronic disorders (106). However, further studies are required to better clarify the involvement of senescent CD8+ T cells during homeostasis and disease.

## CD8+ T cells during ageing

CD8+ T cell compartment is essential in providing long-term immune protection, but its composition may be altered during aging (26, 28, 89). Naïve CD8+ T cells are mainly located in the blood, spleen and lymph nodes, where they can respond to new antigens, while memory CD8+ T cells are found predominantly in tissues such as lung, gut, and brain to rapidly protect against potential infections (11, 22, 26, 60, 77, 107). Infancy is characterized by the egress of a large number of naïve CD8+ T cells from the thymus, which then differentiate into memory cells upon antigen exposure (26, 28, 108). Differently, aging is associated with immunosenescence, resulting in a decrease in the number of naïve and memory CD8+ T cells and an increase of senescent CD8+ T lymphocytes, particularly in the blood and blood-rich sites, such as spleen and lungs (26, 28, 60, 77, 82, 109). During aging, the production of naïve CD8+ T lymphocytes significantly decreases due to age-related thymic involution. This process also leads to changes in the phenotype of CD8+ T cells, such as an increased generation of self-reactive T cells (110, 111), potentially explaining the higher occurrence of autoimmune disorders in older individuals (112).

Moreover, the reduced adaptive immune response during aging promotes innate immunity responses and the release of pro-inflammatory mediators, leading to a state of low-grade systemic inflammation, creating a vicious cycle that further contributes to immunosenescence (113–115). Inflammageing also causes the accumulation of T_EX_ and senescent CD8+ T cells in various organs, where they can make up to 60% of all CD8+ T cells, contributing to significant immune alterations during aging (115, 116). This overall decline in T cell functionality, combined with an increased apoptotic rate of T lymphocytes, can lead to the development of age-related diseases, including neurodegeneration (114). Accordingly, it was recently demonstrated that accumulation of CD8+ T cells in the CNS of aged mice drives axonal degeneration and contributes to age-related cognitive and motor decline through the release of the cytotoxic molecule granzyme B (20). In addition, it was shown that clonally expanded INF-γ-producing CD8+ T cells infiltrate old neurogenic niches in the healthy brain, inhibiting the proliferation of neural stem-cells, potentially contributing to age-related deterioration of brain functions (117). These data suggest that targeting CD8+ CNS-associated T cells in older adults might mitigate aging-related decline of brain structures and functions.

Despite these evidences showing that alterations in CD8+ T lymphocyte characterize ageing and sustain neurodegeneration, it is still debated whether these are the cause or consequence of age-related micro-environment perturbations. Recent findings suggest that reduced extrinsic nutritional availability of glucose, amino-acids, and lipids in older tissues may negatively affect CD8+ T cell functioning. In support of this, metabolic alterations are considered among the main differences between young and old T lymphocytes (113, 118). Thus, the immune changes considered as characteristics of ageing could be instead viewed as the manifestation of elderly-related environmental interferences, which can be modulated by lifestyle factors. Overall, age-related T cell dysfunctions can be regarded as alterations potentially mitigated by a nutrition- and exercise-based approach to improve human health and longevity (118, 119).

## CD8+ T cells in brain diseases

Immunosenescence and inflammageing play a role in patients with neurodegenerative and neuroinflammatory disorders such as AD, PD, and MS, in which the well-balanced inflammatory and anti-inflammatory equilibrium is lost, leading to a prolonged and uncontrolled state of chronic low-grade inflammation (114, 120). In addition, senescent CD8+ T lymphocytes were also detected in the brain of patients affected by LE-induced TLE and Sus, suggesting common pathogenic mechanisms underlying different brain disorders (13, 18).

### Alzheimer’s disease

AD is a progressive neurodegenerative disorder characterized by neuronal death and accumulation of amyloid beta (Aβ) deposits and hyperphosphorylated tau protein in the brain, leading to memory loss and cognitive impairment (121). To date, approximately 35 million people worldwide have been affected by AD, making this disease the most common cause of dementia (122). Approximatively 75% of AD subjects are 75 years old or older, indicating a strong correlation between the development of AD and aging (123). Moreover, a shorter telomere length in CD8+ T cells was correlated to a greater AD severity, together with a lower CD28 expression and an increase of cytotoxic molecules and sensitivity to apoptosis, suggesting the presence of dysfunctional CD8+ T cells in AD (124, 125). The majority of studies performed in AD showed changes in the overall CD8+ T cell population in mice developing amyloid-related pathology, that may not closely reflect the human condition in which tau pathology also represents a disease hallmark (8, 10, 15, 17). Indeed, the association between tau pathology and CD8 T cells was previously suggested, although more recent studies found no correlation between tau hyperphosphorylation and the presence of cytotoxic T cells (15, 126, 127).

**Figure 2:**
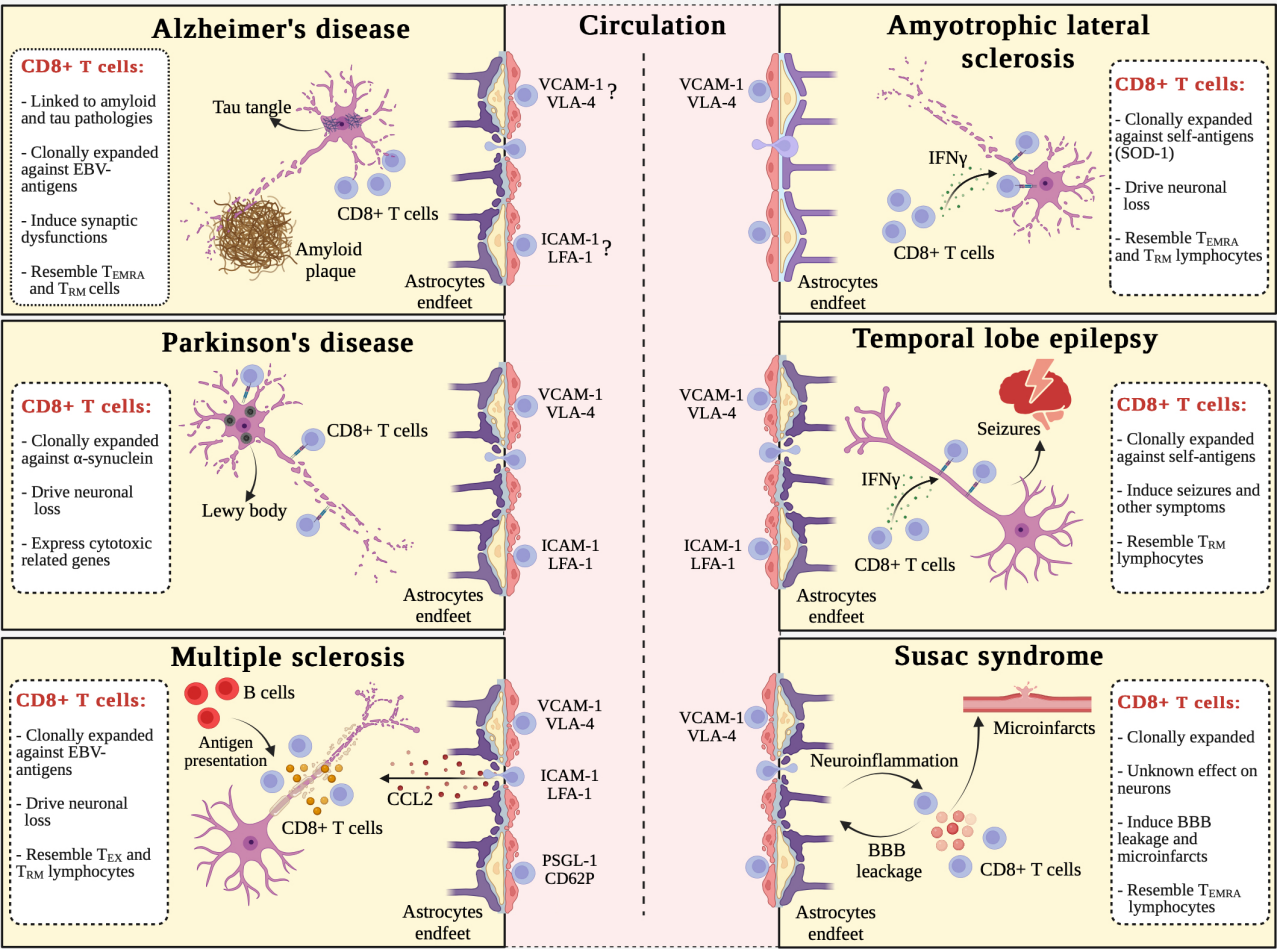
Commonalities and differences in CD8+ T cell-driven immune responses in brain disorders. Alzheimer’s disease (AD), Parkinson’s disease (PD), multiple sclerosis (MS), amyotrophic lateral sclerosis (ALS), limbic encephalitis (LE)-induced temporal lobe epilepsy (TLE), and Susac syndrome (Sus) are all characterized by the migration of circulating CD8+ T lymphocytes into the CNS. The following molecular pathways drive the extravasation of CD8+ T cells in these disorders: (i) VCAM-1/VLA-4 adhesion pathway contributes to all diseases discussed in this review; (ii) ICAM-1/LFA-1 adhesion pathway has a role in PD, MS, and LE-induced TLE; and (iii) PSGL-1/CD62P adhesion pathway is involved only in MS. Once in the CNS, CD8+ T lymphocytes clonally expanded against non-self (AD, MS, and Sus) or self (PD, ALS, and LE-induced TLE) antigens: (i) induce synaptic dysfunctions in AD; (ii) drive neuronal loss in PD, MS, and ALS; (iii) support seizures and other epilepsy-related symptoms in LE-induced TLE; and (iv) promote blood-brain barrier (BBB) leakage, neuroinflammation, and microinfarcts damaging endothelial cells in Sus. CD8+ T cell-driven cellular alterations are induced by direct cytotoxicity (TCR-MHC-I binding) in PD, ALS, and LE-induced TLE, as well as soluble factors, including granzymes, in MS and Sus. In addition, two district features characterize MS: (i) B cells present Epstein Barr-derived (EBV) antigens to T cells; and (ii) CCL2 is crucially involved in the homing of blood-derived CD8+ T lymphocytes to the inflamed CNS. ALS is apparently the only disorder not yet associated to BBB dysfunction. CD8+ T cells from patients with AD, ALS, and Sus show a T_EMRA_ senescent-like phenotype, while in MS CD8+ T cells have an “exhausted” phenotype. Except for PD, CD8+ T lymphocytes from all discussed CNS disorders, have tissue resident memory (T_RM_) traits. Created with Biorender.com.

The heterogenicity of the CD8+ T compartment has been only recently studied in AD, showing clonally expanded CD8+ T_EMRA_ cells with cytotoxic potential in the cerebrospinal fluid (CSF) of AD individuals (6). Surprisingly, T_EMRA_ lymphocytes were not clonally expanded against AD-specific antigens such as Aβ peptides or tau protein, but they were reactive in the presence of Epstein-Barr virus (EBV) antigens (6) (**Figure 2**). These data do not provide a causal link between EBV infection and AD, but suggest that senescence of CD8+ T lymphocytes may play a role in AD (128). Furthermore, the absence of clonal expansion toward disease-specific antigens suggests that CD8+ T cell trafficking into the CNS could be a stochastic phenomenon (6, 17). Accordingly, the damage of CNS barriers previously described in AD can favor nonspecific CD8+ T cells brain invasion from the blood circulation (129) (**Figure 2**). The increased expression of lymphocyte function-associated antigen 1 (LFA-1) integrin on infiltrating total T cells in the AD brain, and the augmented expression of *Itgb2* gene, encoding for the CD18 subunit of LFA-1 molecule, in the hippocampus of a mouse model of tauopathy compared to controls suggest a role for LFA-1-intercellular adhesion molecule-1 (ICAM-1) interactions in the migration of CD8+ T cells in the CNS during AD (8, 126). This is also supported by the increased expression of ICAM-1 detected on brain endothelial cells of mice with AD-like disease and higher levels of soluble ICAM-1 in the plasma from AD subjects compared to controls (3, 130).

Although ablation of CD8+ T cells in AD mice did not reduce Aβ deposition and cognitive deficits in APP-PS1 mice at later disease stages, recent studies suggested that CD8+ T cells migrate in the brain parenchyma in subjects with AD and its mouse models (15, 127) (**Figure 2**). The phenotype of CD8+ T lymphocytes in aged and AD transgenic mice have a T_RM_ gene signature, but the characterization is still in its infancy (17, 20) (**Figure 2**). In summary, existing literature suggests CD8+ T cell heterogeneity in AD, highlighting a potential role for T_EMRA_ and T_RM_ cells subsets in the pathogenesis of this disorder. However, the involvement of CD8+ T cells in AD is still unclear and more studies are needed to identify the molecular mechanisms mediating a potential CD8+ T cell-dependent damage.

### Parkinson’s disease

PD is the second most common form of neurodegenerative disorder, affecting more than 10 millions people around the world, with an increased prevalence in the aged population (131). The main neuropathological hallmark of the disease is the presence α-synuclein (α-syn) aggregates, referred to as Lewy bodies, and Lewy neurites, observed in neuromelanin-containing neurons of the substantia nigra (SN) (132). This is associated with the classical PD symptoms of bradykinesia, rest tremor, muscular rigidity, motor and cognitive alterations, as well as autonomic dysfunctions (132). Similarly to AD, the initiators of the pathogenic cascade leading to neuronal death and to the related neurological alterations are presently poorly understood in PD.

Recently, a study conducted in PD patients provided novel insights into the T cell-mediated adaptive immune responses by performing single-cell transcriptome and TCR sequencing, revealing a continuous progression of CD8+ T cells from a central memory to a terminal effector phenotype (133). In addition, previous data showed that CD8+ T cells are increased in the substantia nigra of diagnosed PD cases and positively correlate with neuronal death (5). Interestingly, CD8+ T cells infiltration is present since early disease stages, preceding neuronal loss and α-syn aggregation, suggesting a role for these cells in disease development (5). Notably, although a direct demonstration is still lacking, CD8+ T cells with a T_RM_ phenotype were detected near altered neurons in the substantia nigra, suggesting that CD8+ T cells may exert cytotoxic functions potentially contributing to the pathological changes in PD (5) (**Figure 2**). Furthermore, a longitudinal case study and analysis of two independent cohorts revealed that elevated α-syn-specific CD8+ T cell responses in the blood were present prior to and after diagnosis of motor PD, and were significantly associated with age (134) (**Figure 2**). These studies suggest that CD8+ T cell infiltration is an early event in PD, paralleling the progression of neuronal death and synucleinopathy, providing insight into new disease mechanisms and early diagnosis in PD. Accordingly, T cells from PD patients do not react to common antigens, but are activated by α-syn-derived antigens, suggesting that the T cell responses observed in PD may be prevalently directed towards autoantigens (135).

Recent data showed a core gene signature for α-syn-reactive CD8+ T lymphocytes in PD, which includes the expression of *CX3CR1*, *CCR5*, *CCR1* pro-inflammatory genes, but also genes expressed in neurons such as *LRRK2*, *LAMP3*, and aquaporin genes, previously associated with PD worsening (136). Interestingly, the increased expression of the leucine-rich repeat kinase 2 (LRRK2) in CD8+ T lymphocytes from PD patients correlated with an increased secretion of pro-inflammatory molecules and cell activation, suggesting that LRRK2 may represent a therapeutic target in PD (137). Accordingly, increased expression of granzymes and perforin 1 in clonally expanded CD8+ T cells are present in the blood and CSF of PD patients (133) (**Figure 2**). Also, the increased cytotoxicity of CD8+ T lymphocytes in PD correlates with a terminal effector phenotype and the expression of proteins involved in cell migration, such as CX3CR1 and the adhesion G protein-coupled receptor G1 (ADGRG1) (133). The migration of CD8+ T lymphocytes into the CNS during PD (5) has been associated with the upregulation of *ITGAM* and *ITGB1* genes, suggesting a role for CD11b and CD29, subunits of LFA-1 and very late antigen-4 (VLA-4) integrins, respectively (133) (**Figure 2**). Moreover, the disruption of the blood-brain barrier (BBB) in PD (138, 139), indicate that vascular phenomena may also contribute to CD8+ T cell infiltration into the CNS (**Figure 2**). Overall, these findings emphasize an increased immune reactivity of CD8+ T cells against CNS antigens in PD, which is associated with an enhanced clonally expanded T cell infiltration and cytotoxicity in the brain, suggesting a circulating origin of α-syn-reactive CD8+ T lymphocytes in the PD brains (133, 135). Future studies exploring the phenotype and functions of CD8+ T lymphocyte subsets in PD could lead to the identification of novel and specific therapeutic targets for this neurodegenerative disease.

### Multiple sclerosis

MS is a chronic inflammatory and autoimmune disorder of the CNS affecting approximatively 2.8 million people worldwide (140). Multifocal inflammatory lesions develop in both brain and spinal cord (SC), resulting in demyelination and neurodegeneration and leading to a progressive decline in motor, sensory, and cognitive functions (141). In most patients, MS is characterized by a relapsing and remitting onset, followed by a chronic, prolonged, and progressive inflammatory state during which the neurological symptoms gradually worse (141). Several studies have shown that CD8+ T lymphocytes predominate in active MS lesions (142–144). Furthermore, CD8+ T cells from in MS lesions are clonally expanded against common antigens and have been probably recruited from the periphery (12, 142, 145) (**Figure 2**). Indeed, it has been demonstrated that blood, CSF and brain CD8+ T cell clones share a high degree of phenotypic similarity further supporting the view that CD8+ T cells invading the MS brain originate from the periphery and contribute to MS progression (144, 145). The homing of clonally expanded CD8+ T lymphocytes to the CNS of MS patients may be further promoted by BBB damage and increased expression of adhesion molecules and chemoattractants on brain endothelial cells (143, 146) (**Figure 2**). Moreover, previous studies have shown a role for platelet and endothelial cell adhesion molecule 1 (PECAM1), P-selectin glycoprotein ligand-1 (PSGL-1), vascular cell adhesion protein-1 (VCAM-1), intracellular adhesion molecule-1 (ICAM-1), VLA-4 and LFA-1 in the migration of CD8+ T cells into the CNS (143, 144, 147, 148) (**Figure 2**). Once infiltrated to the CNS, CD8+ T cells express higher levels of cytotoxic molecules (19, 143, 144), thus suggesting their detrimental role in the progression of MS-related neurological alterations. Indeed, CD49d+ CD8+ T lymphocytes invading the MS brain exhibited a pro-inflammatory effector phenotype, expressing CD137 and CD95L, as well as inhibitory receptors TIM-3 and PD-1 on their surface, in addition to the transcriptional factor EOMES (12, 14, 19, 143) (**Figure 2**). Importantly, an increased production of granzyme B by CD8+ T cells in MS lesions, has been suggested to contribute to neuronal alterations (143, 144) (**Figure 2**). In line with these findings, in early MS brain lesions the majority of CD8+ T lymphocytes express CD69, but not CD103, and were shown to contain granzyme B (12). Although a link has been suggested between T cell exhaustion and the progression of chronic neuroinflammation in MS, the contribution of T_EX_ CD8+ T lymphocytes in MS course is unclear (149). EBV infection is now considered a key environmental factor for chronic CNS inflammation during MS and CD8+ T cells are clonally expanded against EBV-derived antigens presented by B cells in MS patients (12). EBV can establish a prolonged latent and intermittent reactivation within B cells (150), potentially resulting in a series of CD8+ T cells immune responses, suggesting that B cells may represent essential players in promoting chronic CD8+ T lymphocyte activity in MS (151). Overall, MS represents the neurodegenerative disease in which CD8+ T cells were studied more into detail, although the molecular mechanisms leading to brain damage are not yet fully understood.

### Amyotrophic lateral sclerosis

ALS is an incurable and devastating neurodegenerative disorder, that causes a progressive degeneration of motor neurons leading to a loss of voluntary muscle control and, in severe cases, to respiratory failure (152). Most commonly, symptoms of ALS appear between the ages of 40 and 70, and several genes were associated to the development of this disease, including superoxide dismutase 1 (*SOD1)* (152–155). The normal function of SOD1 protein is to protect cells from oxidative damage, and its alterations lead to increased oxidative stress and mitochondrial dysfunctions (156). In addition, oxidative stress levels are substantially increased by neuroinflammation, which has been recently included among the hallmarks of ALS (157, 158).

A role for CD8+ T lymphocytes in SOD1-associated ALS form was recently suggested (159–161). Particularly, peripheral CD8+ T cell ablation has been shown to increase motoneuron survival in a mouse model of ALS, whereas *in vitro* studies showed that SOD-1 expressing CD8+ T lymphocytes recognize and selectively kill motoneurons via binding MHC-I molecules expressed on these cells, suggesting a possible autoimmune origin for ALS (159) (**Figure 2**). Moreover, activated CD8+ T lymphocytes expressing mutant SOD-1 produce high levels of IFNγ and eliminate a subset of motoneurons in ALS through an antigen restricted, MHC-I-dependent cytotoxic pathway, suggesting a neurotoxic role for self-reactive CD8+ T cells in ALS (159) (**Figure 2**). These data were supported by studies performed in ALS patients showing an increased activation of peripheral and intrathecal CD8+ T cells, with the activation status of CD8+ lymphocytes in the blood being higher in ALS compared to MS and dementia, further suggesting a role for cytotoxic lymphocytes in ALS (160).

Clonally expanded T_EMRA_ CD8+ T cells originating from the circulation have been reported in the CNS of *Setx* knock-in (KI) mice developing ALS4-like disease, as well as in ALS4 patients, who typically have mutations on senataxin (*SETX*) gene (18) (**Figure 2**). These data point to T_EMRA_ cells as negative contributors during ALS and are further supported by a recent retrospective study demonstrating higher frequencies of senescent-like T cells in ALS individuals, suggesting that lymphocyte senescence may drive disease progression (162). Furthermore, the immunophenotyping of T_EMRA_ CD8+ T lymphocytes detected in the CNS of *Setx* KI mice revealed a CD49d+ PD-1+ CD103- profile (18), which is consistent with their peripheral origin in ALS4-like mice, suggesting pathological changes of the T_RM_ CD8+ T cell compartment similar to those observed in MS (12) (**Figure 2**). Nevertheless, differently from MS, the severity of ALS does not appear to be correlated with BBB leakage, suggesting that the infiltration of CD8+ T cells into the CNS of ALS patients and animal models could represent a tightly regulated process, rather than being favored by stochastic events (163, 164) (**Figure 2**). Recent data obtained in two ALS mouse models showed that blocking α4-integrins reduces the migration of peripheral immune cells into the CNS and decreases IFNγ, which is primarily produced by CD8+ T lymphocytes and NK cells, further supporting a role for peripheral cytotoxic T cells in ALS (159, 161, 165). However, more research is needed to identify the molecular mechanisms governing the extravasation of CD8+ T cells into the CNS during ALS.

### Limbic encephalitis-induced temporal lobe epilepsy

Limbic encephalitis (LE)-induced temporal lobe epilepsy (TLE) is a rare form of epilepsy characterized by different types of epileptic seizures, including focal seizures, secondarily generalized seizures, and status epilepticus (166). This subtype of encephalitis is also characterized by a potent inflammatory reaction predominantly against neurons in the grey matter of the medial temporal lobes of the brain, leading to the generation of recurrent seizures (167, 168). Previous studies associated neuroinflammation, BBB dysfunction and leukocyte migration to the induction of seizures (169–172).

LE is triggered by an autoimmune response favored by various underlying causes, including viral infections and paraneoplastic syndromes (173–175). Indeed, the presence of autoantibodies directed toward neuronal surface antigens (NSAbs) was well described in LE patients (176–180). Moreover, it was suggested that IFNγ-producing CD8+ T lymphocytes promote MHC-I upregulation on LE neurons, thus supporting a neuron-directed CD8+ T cell attack, similar to the immune reactivity demonstrated in ALS (16, 181) (**Figure 2**). CD8-mediated neurotoxicity has been suggested to contribute to neuronal excitability and acute seizure generation, promoting psychiatric disturbances, memory impairment and behavioral changes (182). Furthermore, it has been hypothesized that chronic neuroinflammation can induce persistent changes in the structural and electrical properties of certain neuronal networks, resulting in the development of chronic spontaneous seizures and epilepsy (16, 181, 182) (**Figure 2**). Notably, infiltration of granzyme B-producing CD8+ T lymphocytes, which may attack and destroy neurons expressing MHC-I, was found among other leukocyte populations in the hippocampi of TLE patients (2, 16, 183–188) (**Figure 2**). Interestingly, cytotoxic CD8+ T cells accumulate in the brain of LE patients, suggesting that self-reactive CD8+ T cells can directly cause neurotoxicity, but can also contribute to the conversion of LE into TLE (178) (**Figure 2**).

Recent studies showed that activated CD44+ ovalbumin (OVA)-specific CD8+ T cells, directed against the “SIINFEKL” chicken peptide (OVA 257-264) expressed exclusively by hippocampal neurons, migrate into the brain and promote seizures (16). Furthermore, CD8+ T lymphocyte infiltration leads to prolonged epileptic activity and memory deficits, suggesting that circulating CD8+ T cells may play a role in the induction and progression of LE-induced TLE (16) (**Figure 2**). Brain invasion by circulating CD8+ T lymphocytes correlate to BBB dysfunction in experimental models of TLE and magnetic resonance imaging revealed BBB alterations after the transfer of OVA-specific CD8+ T cells, followed by a size reduction and degeneration of the hippocampus (2, 16, 169) (**Figure 2**). Moreover, brain-invading CD8+ T lymphocytes showed an increased expression of CD69 surface marker, suggesting that these cells may acquire a T_RM_ phenotype once migrated into the brain (16), similarly to what has been previously described in MS (12). This may be supported by previous studies showing that ICAM-1 and VCAM-1 are upregulated in TLE hippocampi and after seizure induction and that preventive blockade of α4 integrins or ICAM-1 adhesion receptor abrogates seizure induction (2, 169). Of note, TLE patients display a T cell activated phenotype in peripheral blood (185). Thus, CD8+ T lymphocytes could migrate into the inflamed brain in TLE using LFA-1 and/or α-4 integrins, as shown in ALS and as previously suggested in patients with MS and epilepsy (189) (**Figure 2**).

### Susac syndrome

Sus is a rare disorder characterized by neuroinflammation and CNS dysfunction, due to focal microangiopathy affecting small and medium size vessels of the brain, retina and the inner ear (190, 191). The etiology of Sus remains largely unknown, and the role of neuroinflammation has only recently started to be addressed. However, the successful use of immunosuppressive and immunoregulatory drugs in Sus cases, support an autoimmune origin for this disease (192, 193). Accordingly, an increased immune cell infiltration into the brain, with the majority of cells being CD8+ T lymphocytes, has been found in Sus patients compared to healthy controls (9, 194). Also, it has been suggested that circulating self-reactive CD8+ T lymphocytes may promote endothelial cell alterations and BBB dysfunction during Sus, potentially leading to microinfarcts and leukocyte transmigration across the BBB (13) (**Figure 2**). Moreover, Sus patients treated with natalizumab, which inhibits lymphocyte migration into the CNS, or mice treated with an antibody blocking anti-α4 integrins, showed a reduction in disease severity compared to controls, further supporting the idea that Sus shares several key immunopathological elements with MS (13) (**Figure 2**). Indeed, VLA-4 neutralization was found to restrict infiltration of CD8+ T lymphocytes into the CNS of EAE mice (143, 195), suggesting this may be the case also in Sus. Despite several common disease mechanism between MS and Sus, the total number of leukocytes detected in the CSF is in the normal range in Sus patients, whereas it is significantly increased in MS cases (13). However, the proportion of CD8+ T cells is selectively and significantly increased in the CSF of Sus subjects, suggesting a role for these cells in Sus (13). Sus patients have a larger proportion of clonally expanded CD57+ CD8+ T_EMRA_ lymphocytes of circulating origin close to disease onset, with lower clonal expansion levels during remission, further supporting a relationship between Sus pathogenesis and CD8+ T cells (13). Moreover, immunophenotyping of CD8+ T lymphocytes in brain of Sus patients revealed a cytotoxic capacity, as evidenced by the higher expression of granzyme B lytic molecule compared to healthy controls (13) (**Figure 2**). Overall, several lines of evidence point to a key role for CD8+ cells in Sus, but more studies are needed to understand how these cells contribute to BBB dysfunction and neuronal damage.

## Conclusions

CD8+ T lymphocytes are adaptive immune cells that, upon antigen recognition, undergo a complex differentiation process (**Figure 1**). In acute inflammatory responses, when antigen is effectively cleared, short-lived effector T cells undergo controlled apoptosis, while long-lived effector T lymphocytes differentiate into memory T cells, thus efficiently resolving the inflammatory reaction. However, during chronic inflammatory conditions, this natural resolution is impaired, and CD8+ T lymphocytes become exhausted or senescent, retaining a neurotoxic potential and contributing to several neurodegenerative diseases. CD8+ T cells, reacting against self and non-self antigens are clonally expanded in all brain disorders discussed in this review: AD, PD, MS, ALS, LE-induced TLE and Sus. It is worth noting that although these disorders may have distinct causes, occurrence rates, and clinical presentations, they share common immunopathological characteristics. These include the circulating origin of CNS-invading CD8+ T lymphocytes, the clonal expansion of CD8+ T cells, and phenotypical traits that resemble senescence (**Figure 2**). In the light of growing evidence suggesting that senescent and exhausted CD8+ T cells contribute to aging and various brain disorders, a promising therapeutic approach for these conditions may be represented by targeting deleterious functions of CD8+ T cells. Indeed, targeting senescent and exhausted CD8+ T cells may create a personalized neuroimmunotherapy, with the ultimate goal to rejuvenate T cells through tailored diagnostic and therapeutic protocols (87, 196). Strategies such as epigenetic modulation and using senolytic compounds to induce apoptosis in senescent and exhausted CD8+ T cells may also be explored. Several studies are ongoing to prove the effectiveness of interventions targeting tissue-damaging senescent cells, which may slow, prevent, and alleviate disorders in preclinical models (197). The development of senolytic small-molecules that can specifically eliminate senescent cells, may represent a promising strategy for treating multiple CD8+ T cell senescent-mediated disorders and age-related conditions in humans (197). Also, the epigenetic modulation of senescent and exhausted CD8+ T cells involving small molecules and biologics to target the molecular pathways involved in developing and maintaining these cell types, can modify the senescence and exhaustion, potentially reversing these deleterious phenotypes (198–200). For example, it has been recently observed that EZH2-expressing T cells are precursors to KLRG1+ effector lymphocytes, while EZH2^LOW^-expressing T cells predominantly produce non-cytotoxic CD103+ CD69+ TRM CD8+ T cells (201). Thus, the silencing or deficiency of the *Ezh2* gene, which mediates these epigenetic modifications in CD8+ T lymphocytes, may be therefore targeted to induce repression of the exhausted phenotype (200). Overall, these approaches may help to reduce the number of neurotoxic CD8+ T cells and potentially mitigate the effects of aging and neuroinflammatory disorders.

## Author contributions

All authors listed have made a substantial, direct, and intellectual contribution to the work and approved it for publication.
